# Dual-/tri-apodization techniques for high frequency ultrasound imaging: a simulation study

**DOI:** 10.1186/1475-925X-13-143

**Published:** 2014-10-11

**Authors:** Jin Ho Sung, Jong Seob Jeong

**Affiliations:** Department of Medical Biotechnology, Dongguk University, Seoul, 100-715 Republic of Korea

## Abstract

**Background:**

In the ultrasound B-mode (Brightness-mode) imaging, high side-lobe level reduces contrast to noise ratio (CNR). A linear apodization scheme by using the window function can suppress the side-lobe level while the main-lobe width is increased resulting in degraded lateral resolution. In order to reduce the side-lobe level without sacrificing the main-lobe width, a non-linear apodization method has been suggested.

**Methods:**

In this paper, we computationally evaluated the performance of the non-linear apodization method such as dual-/tri-apodization focusing on the high frequency ultrasound image. The rectangular, Dolph-Chebyshev, and Kaiser window functions were employed to implement dual-/tri-apodization algorithms. The point and cyst target simulations were conducted by using a dedicated ultrasound simulation tool called Field-II. The center frequency of the simulated linear array transducer was 40 MHz and the total number of elements was 128. The performance of dual-/tri-apodization was compared with that of the rectangular window function focusing on the side-lobe level and the main-lobe widths (at -6 dB and -35 dB).

**Results:**

In the point target simulation, the main-lobe widths of the dual-/tri-apodization were very similar to that of the rectangular window, and the side-lobe levels of the dual-/tri-apodization were more suppressed by 9 ~ 10 dB. In the cyst target simulation, CNR values of the dual-/tri-apodization were improved by 41% and 51%, respectively.

**Conclusions:**

The performance of the non-linear apodization was numerically investigated. In comparison with the rectangular window function, the non-linear apodization method such as dual- and tri-apodization had low side-lobe level without sacrificing the main-lobe width. Thus, it can be a potential way to increase CNR maintaining the main-lobe width in the high frequency ultrasound imaging.

## Background

In the diagnostic ultrasound imaging by using an array transducer, a beamforming technique is commonly used for electrical transmit/receive focusing, beam steering, and dynamic focusing. A conventional delay and sum (DAS) beamforming enhances the signals from the selected location and reduces the signals from undesired direction by compensating arrival time of received signals. Although the conventional DAS beamforming can increase the energy of the ultrasound beam at the focal point, the side-lobe level also increases resulting in degraded contrast to noise ratio (CNR) [1].

In order to reduce the side-lobe level, linear apodization methods by using various window functions were developed. The window functions realized by changing the amplitude of the signals can effectively suppress the side-lobe level. However, the main-lobe width is increased resulting in reduced lateral resolution.

To solve this problem, some researchers have proposed several methods such as constrained least squares (CLS), non-linear apodization, and dual-apodization with cross-correlation (DAX) methods [2–8]. Among them, the non-linear apodization method especially multi-apodization technique can be easily implemented and the processing time is short compared to other methods. However, the performance was mainly demonstrated by impulse response plotting without applying to ultrasound imaging [4].

In this study, we computationally evaluated the performance of dual-/tri-apodization focusing on the high-frequency ultrasound imaging since it suffers from high side-lobe level and noise components. A Field-II, a dedicated ultrasound simulation tool, was employed for this demonstration. The rectangular, Dolph-chebyshev, and Kaiser window functions were chosen for dual-/tri-apodization. The point and cyst target simulations were conducted and subsequently the main-lobe width, the side-lobe level, and CNR value were measured. The simulation results show that dual-/tri-apodization can effectively suppress the side-lobe level and increase CNR without degrading lateral resolution in the high-frequency ultrasound imaging.

## Methods

### A. Selection of window functions

The rectangular, Dolph-Chebyshev, and Kaiser window functions were chosen for dual- and tri-apodization since the rectangular window function has the narrowest main-lobe width, and other window functions can control the main-lobe width and the side-lobe level by adjusting parameters. Before explaining the principle of dual-/tri-apodization, the features of used window functions were discussed.

It has been well known that the rectangular window function called uniform window function has the highest side-lobe level and the narrowest main-lobe width compared to other window functions. The side-lobe level of the rectangular window function is -13 dB, and -6 dB main-lobe width is 1.21 bins. The rectangular window function in time domain is defined by equation (1) [9].
1

where N is the total number of samples and *n* is the sample number in time domain. The discrete Fourier transform (DFT) of the rectangular window function can be defined by
2

where *k* is the sample number in frequency domain.

The Dolph-Chebyshev window function was developed as one of the antenna design techniques and has the control parameter for the main-lobe width and the side-lobe level. The Dolph-Chebyshev window function in time domain can be written as
3

where *W*_*D-C*_*(k)* is the DFT of the Dolph-Chebyshev window function and it is defined by equation (4) [9–13].
4

*β* is defined as
5

and
6

where *α* in (5) is the log of the ratio of main-lobe level to side-lobe level and (-1)^*k*^ in (4) expresses the shifted origin in the time domain signal.

The Kaiser window function was come from the zero-order modified Bessel function. Similar to the Dolph-Chebyshev window function, the Kaiser window function has the control parameter *α*_*K*_ which determines the main-lobe width and the side-lobe level. Note that the subscript *K* is named after the Kaiser window function. The Kaiser window function in time domain is defined as [14]
7

where *α*_*K*_ is a control parameter and *I*_0_(*X*) is the zero-order modified Bessel function defined as
8

The DFT of the Kaiser window function is defined as
9

### B. Principle and effect of dual-/tri-apodization

Since the rectangular window function has the narrowest main-lobe width, and the Dolph-Chebyshev and Kaiser window functions have the control parameter which can be optimized depending on applications, we chose the rectangular, Dolph-Chebyshev, and Kaiser window functions for dual-/tri-apodization. The rectangular and Dolph-Chebyshev window functions were used for dual-apodization. In dual-apodization method, the rectangular and Dolph-Chebyshev window functions were applied to input signal making two different output signals. After that, those output signals were normalized by themselves and minimum value of them was selected. Consequently, the main-lobe width and the side-lobe level of the dual-apodization method were identical to the main-lobe width of the rectangular window function and the side-lobe level of the Dolph-Chebyshev window function, respectively. As a similar way, the rectangular, Dolph-Chebyshev, and Kaiser window functions were used for tri-apodization. Each singular window function was applied to input signal respectively making three different output signals, and the same procedure in the dual-apodization was conducted. As a result, tri-apodization also had narrow main-lobe width identical to that of the rectangular window function, and had low side-lobe level affected by the Dolph-Chebyshev and Kaiser window functions. In this paper, we chose control parameters of the Dolph-Chebyshev (*α*) and Kaiser (*α*_*K*_) window functions as 2.5 and 2.5/π, respectively, to optimize the performance of the dual- and tri-apodization.

**Figure 1:**
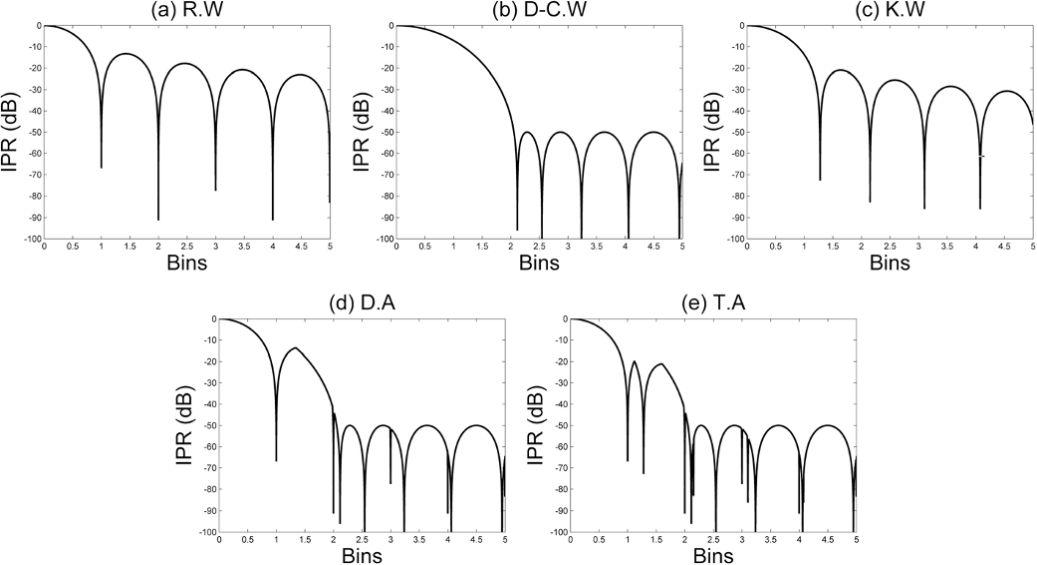
**Impulse responses (IPR) of the singular window functions and dual-/tri-apodization.** Impulse responses (IPR) of **(a)** rectangular window function (R.W), **(b)** Dolph-Chebyshev window function (D-C.W), **(c)** Kaiser window function (K.W), **(d)** dual-apodization (D.A) using rectangular and Dolph-Chebyshev window functions, and **(e)** tri-apodization (T.A) using rectangular, Dolph-Chebyshev, and Kaiser window functions. Bin is a spectrum sample.

To show the theoretical features of the singular window functions and dual-/tri-apodization, their impulse responses (IPR) were obtained as shown in Figure 1. Figure 1(a) shows IPR of the rectangular window function. The -6 dB main-lobe width was 1.21 bins and the highest side-lobe level was -13 dB. Figure 1(b) shows IPR of the Dolph-Chebyshev window function with *α* = 2.5 to decrease near field side-lobe level of dual-apodization by -50 dB and to obtain 1.85 bins main-lobe width at -6 dB.

In the ultrasound image, the side-lobe level should be less than -40 dB because the ultrasound image contrast is determined by the lateral point response at -40 dB [15]. Furthermore, the near field side-lobe level of dual-apodization can be suppressed by the selection of minimal value procedure. Additionally, that is affected by the first cross point between the rectangular and Dolph-Chebyshev window responses. Therefore, considering -40 dB side-lobe level criterion for contrast, we chose the control parameter of the Dolph-Chebyshev window function, *α*, as 2.5.

Figure 1(d) shows IPR of dual-apodization and it had 1.21 bins main-lobe width at -6 dB, which is identical to that of the rectangular window function. The highest side-lobe level of dual-apodization is slightly lower than that of the rectangular window function due to the effect of the main-lobe width of the Dolph-Chebyshev window function. The rest of side-lobe level was -50 dB due to the side-lobe level of the Dolph-Chebyshev window function.

To more suppress the first side-lobe level of dual-apodization, we employed the Kaiser window function and subsequently realized tri-apodization as shown in Figure 1(e). The main-lobe width of the Kaiser window function can exist between the rectangular and Dolph-Chebyshev window functions, and the first side-lobe level can be lower than dual-apodization depending on a control parameter. When we choose *α*_*K*_ =2.5/π, the highest side-lobe level of the Kaiser window function was -21 dB and -6 dB main-lobe width was 1.43 bins (Figure 1(c)). Those specifications contribute the improved performance of the tri-apodization compared to the rectangular function. The main-lobe width was identical, the first side-lobe level was 7 dB low, and the harmonic side-lobe level was -50 dB.

### C. Specification of B-mode simulation

**Figure 2:**
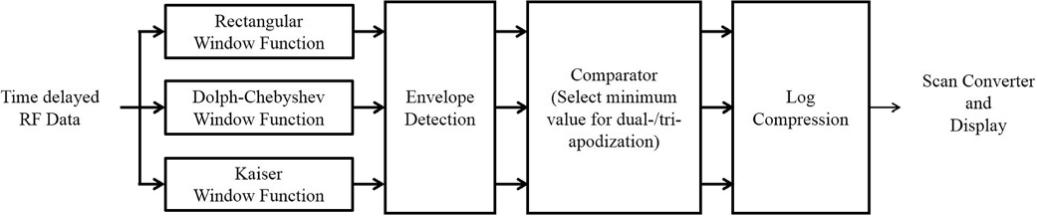
**Block diagram of dual-/tri-apodization algorithms for B-mode image.**

After examining the features of the singular window functions and dual-/tri-apodization, we evaluated the performance of dual-/tri-apodization for targeting the ultrasound B-mode (Brightness-mode) image. By using Field-II and MATLAB program, the singular window functions and dual-/tri-apodization were applied to the point target and cyst target B-mode simulations. The Field-II program capable of providing transmit/receive beam pattern has been widely used for ultrasound imaging [16]. Figure 2 shows a block diagram of dual-/tri-apodization for B-mode imaging. The singular window functions were applied to the received radio frequency (RF) data respectively and each image was normalized by itself after envelope detection to compare different images. After that, minimum value of images was chosen at each spatial location in the comparator stage. Before display of final images of dual-/tri-apodization, the log compression was conducted.

**Figure 3:**
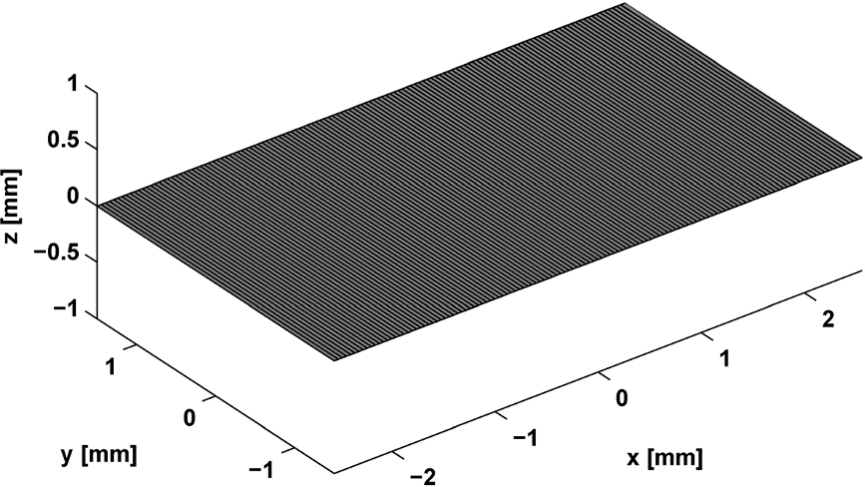
**Aperture of the 128 element linear array transducer.**

In the point target simulation, five point targets were located at 2.5 mm, 3 mm, 3.5 mm, 4 mm, and 4.5 mm in the axial direction. Subsequently, the cyst target simulation was conducted by using the cyst with 1 mm diameter at 3.5 mm depth. The simulation parameters are described in Table 1. The input signal was a sinusoidal wave with 2 cycles. A 128 element 40 MHz linear array transducer was designed as shown in Figure 3, and the number of elements in subaperture was 32. The element pitch was 40 μm and transmit focal length was 3.5 mm. To evaluate the effects of the singular window functions, and dual-/tri-apodization, received data were applied to each method during the receive beamforming to obtain a B-mode image. The performance of each method in the point target simulation was evaluated by the main-lobe widths at the -6 dB and -35 dB, and the side-lobe level. CNR value was measured in the cyst target simulation.

**Table 1:** **Simulated parameters of 128 element linear array transducer**

Parameter	Value
Total Number of Elements	128
Number of Elements in Subaperture	32
Number of Scanlines	128
Center Frequency [MHz]	40
Element Pitch [μm]	40
Speed of Sound [m/s]	1500
Transmit Focus [mm]	3.5

## Simulation results

### A. Point target B-mode simulation

**Figure 4:**
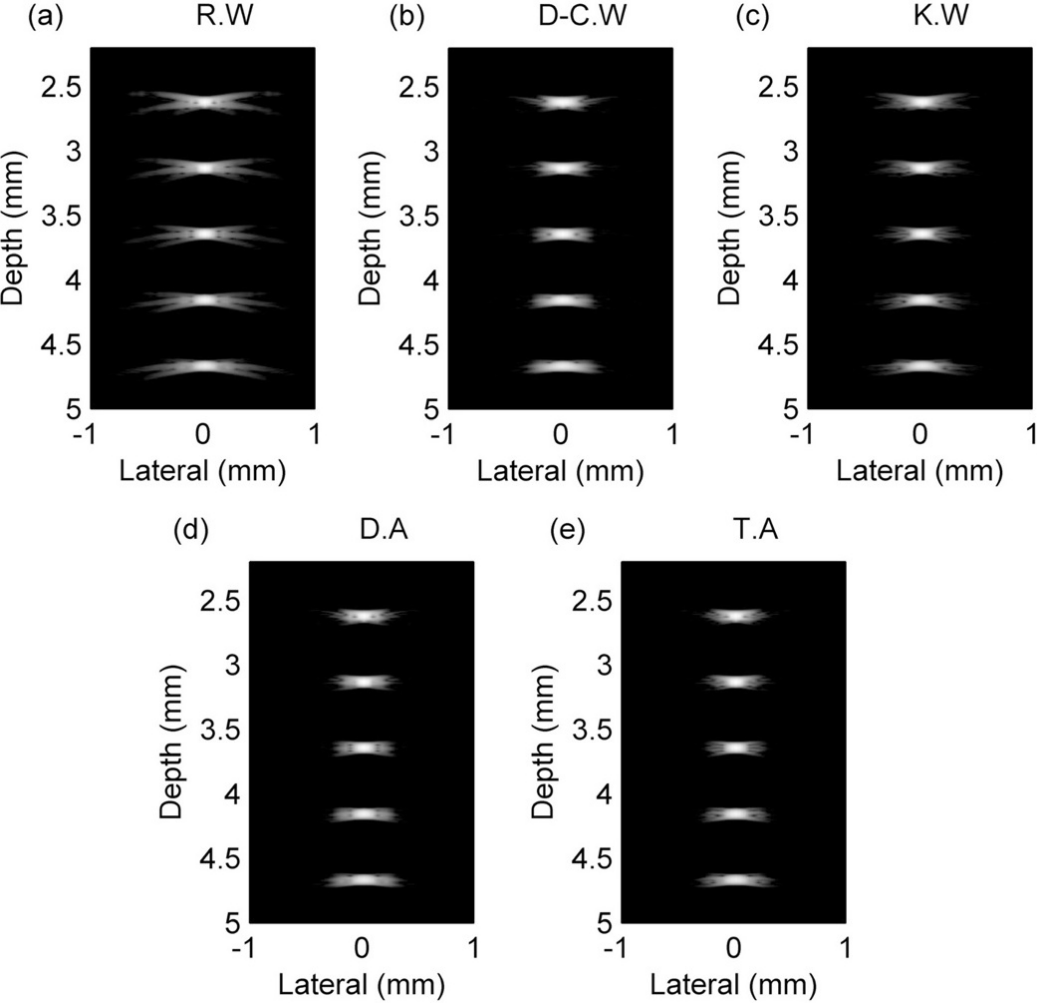
**Point target simulation results of the singular window functions and dual-/tri-apodization.** Point target simulation results of **(a)** rectangular window function (R.W), **(b)** Dolph-Chebyshev window function (D-C.W), **(c)** Kaiser window function (K.W), **(d)** dual-apodization (D.A) using rectangular and Dolph-Chebyshev window functions, and **(e)** tri-apodization (T.A) using rectangular, Dolph-Chebyshev, and Kaiser window functions.

Figure 4 shows B-mode image of the point target simulation with 60 dB dynamic range. The third target was located in the focal point, and the singular window functions and dual-/tri-apodization were respectively applied to the data in receive beamforming. The image of the rectangular window function (Figure 4(a)) shows severe side-lobe artifact, and images of the Dolph-Chebyshev and Kaiser window functions (Figure 4(b), (c)) show blurred target boundary due to the expanded main-lobe width. Figure 4(d), (e) show images of dual-/tri-apodization and they show relatively narrow main-lobe width and low side-lobe level. To evaluate the effect of each method, main-lobe widths at -6 dB and -35 dB, and side-lobe level were calculated from the lateral beam projected data. In order to obtain side-lobe value, the region of interest (ROI) was defined by 0.3 mm ~ 0.4 mm for each target in the lateral direction.

**Figure 5:**
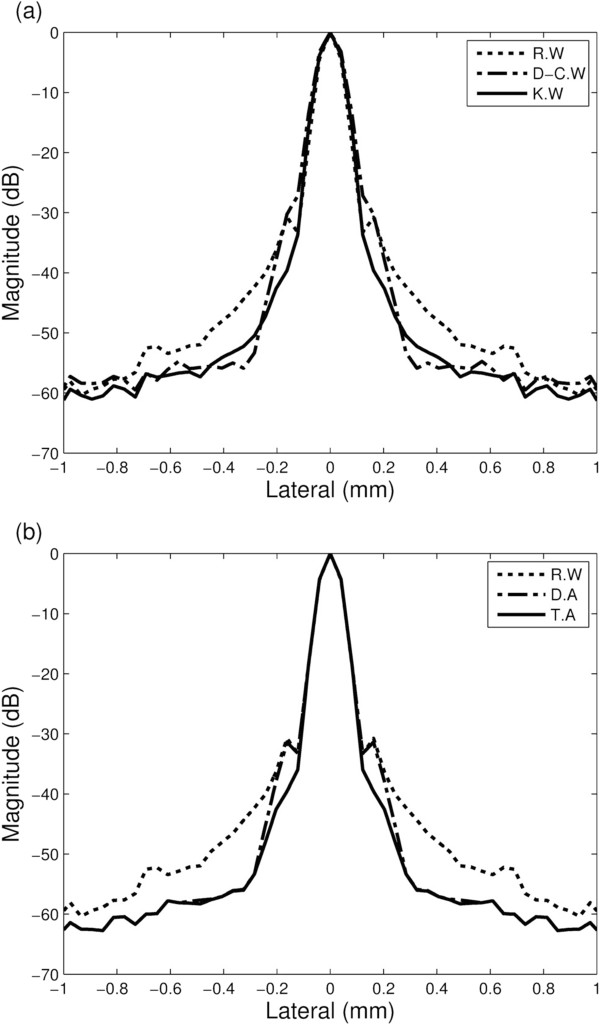
**Lateral beam projections of the singular window functions and dual-/tri-apodization.** Lateral beam projections of **(a)** rectangular window function (R.W), Dolph-Chebyshev window function (D-C.W), and Kaiser window function (K.W), and **(b)** rectangular window function (R.W), dual-apodization (D.A) using rectangular and Dolph-Chebyshev window functions, and tri-apodization (T.A) using rectangular, Dolph-Chebyshev, and Kaiser window functions at the focal point. Note that the rectangular window function was used for reference window to compare the performance of several window functions and D.A/T.A.

To get lateral beam projected data, we set the range in the axial direction enough to include the side-lobe of the each single point target. Since the location where the side-lobe level of each singular window function is maximal is different, dual- and tri-apodization can have lower side-lobe level than the singular window functions. Figure 5(a) shows lateral beam projections of the singular window functions, and Figure 5(b) shows lateral beam projections of the rectangular window function and dual-/tri-apodization at the focal point. The -6 dB main-lobe width of the singular window functions were 90.4 μm, 103.2 μm, and 96.3 μm, in order of the rectangular, Dolph-Chebyshev, and Kaiser window functions. In the dual-/tri-apodization, those main-lobe widths were measured as 91.1 μm similar with that of the rectangular window function. It shows that dual-/tri-apodization have slightly broader main-lobe width than that of the rectangular window function because magnitude of the target in each image is different. That is due to the simulation error, however, its effect is not critical. The results of -35 dB main-lobe width were 386.7 μm, 375.3 μm, 261.5 μm, 370.0 μm, and 239.2 μm in order of the rectangular, Dolph-Chebyshev, Kaiser window functions, dual-apodization, and tri-apodization. It shows that -35 dB main-lobe widths of dual- and tri-apodization are narrower than that of the rectangular window, and thus shows that dual-/tri-apodization can maintain narrow main-lobe width.

At the focal point, the side-lobe level of the rectangular window function was -43 ~ -48 dB, and the Dolph-Chebyshev and Kaiser window functions were -54 ~ -56 dB and -51 ~ -54 dB, respectively. Dual-/tri-apodization had side-lobe level of -54 ~ -57 dB. The point target simulation shows that dual- and tri-apodization can effectively reduce the side-lobe level maintaining the main-lobe width. The -6 dB and -35 dB main-lobe widths were summarized in Tables 2 and 3, and the side-lobe levels were shown in Table 4.

**Table 2:** **-6 dB Main-lobe widths of the singular window functions, and dual-/tri-apodization**

	Rectangular window [μm]	Dolph-Chebyshev window [μm]	Kaiser window [μm]	Dual-apodization [μm]	Tri-apodization [μm]
1st target	90.7	132.2	101.2	91.5	91.5
2nd target	91.2	103.4	93.1	91.2	91.2
3rd target	90.4	103.2	96.3	91.1	91.1
4th target	102.8	123.3	112.0	103.8	103.8
5th target	124.5	163.9	141.2	126.0	126.0

**Table 3:** **-35 dB Main-lobe widths of the singular window functions and dual-/tri-apodization**

	Rectangular window [μm]	Dolph-Chebyshev window [μm]	Kaiser window [μm]	Dual-apodization [μm]	Tri-apodization [μm]
1st target	486.1	351.0	367.7	351.0	331.6
2nd target	386.6	350.1	298.2	350.1	298.2
3rd target	386.7	375.3	261.5	370.0	239.2
4th target	482.3	453.0	349.4	453.0	350.8
5th target	624.9	537.3	463.1	537.3	464.2

**Table 4:** **Side-lobe levels of the singular window functions and dual-/tri-apodization**

	Rectangular window [dB]	Dolph-Chebyshev window [dB]	Kaiser window [dB]	Dual-apodization [dB]	Tri-apodization [dB]
1st target	-39 ~ -45	-46 ~ -52	-47 ~ -51	-49 ~ -54	-52 ~ -55
2nd target	-44 ~ -48	-53 ~ -57	-49 ~ -54	-53 ~ -57	-53 ~ -57
3rd target	-43 ~ -48	-54 ~ -56	-51 ~ -54	-54 ~ -57	-54 ~ -57
4th target	-39 ~ -45	-48 ~ -51	-47 ~ -50	-48 ~ -54	-50 ~ -54
5th target	-35 ~ -40	-40 ~ -48	-41 ~ -46	-40 ~ -48	-42 ~ -49

### B. Cyst target B-mode simulation

For more realistic feasibility study by measuring the CNR value, the cyst target simulation was conducted. Because a cyst has a lot of scatterers, its property is similar to biological tissue. A diameter of the simulated cyst was 1 mm and we evaluated the performance of dual-/tri-apodization by calculating CNR value. Note that CNR represents the ability to distinguish targets from the other tissue or background, and CNR can be calculated by using the formula written below [17].
10

where *S*_*i*_ is the mean brightness value of the cyst target inside, *S*_*o*_ is the mean brightness value of the cyst target outside, *σ*_*i*_ is the variance of the cyst target inside, and *σ*_*o*_ is the variance of the cyst target outside.

**Figure 6:**
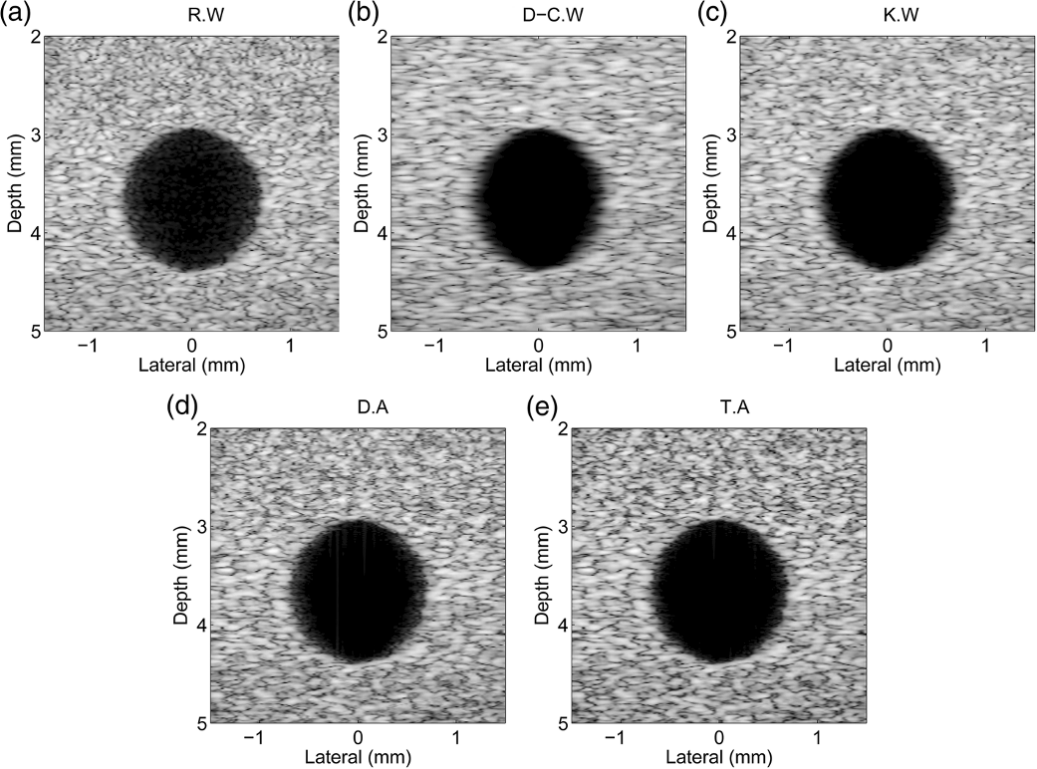
**Cyst target simulation results of the singular window functions and dual-/tri-apodization.** Cyst target simulation results of **(a)** rectangular window function (R.W), **(b)** Dolph-Chebyshev window function (D-C.W), **(c)** Kaiser window function (K.W), **(d)** dual-apodization (D.A) using rectangular and Dolph-Chebyshev window functions, and **(e)** tri-apodization (T.A) using rectangular, Dolph-Chebyshev, and Kaiser window functions.

Figure 6 shows the B-mode cyst image with 60 dB dynamic range. Figure 6(a) shows the image of the rectangular window function. It had small speckle pattern and clear edge compared with images of the Dolph-Chebyshev and Kaiser window functions (Figure 6(b), (c)) due to the narrowest main-lobe width of the rectangular window, but also had the lowest CNR value 2.58 because of the highest side-lobe level. On the contrary, the image of the Dolph-Chebyshev window function had large speckle pattern but had the highest CNR value 4.26 because of expanded main-lobe width and the lowest side-lobe level. Figure 6(c)-(e) are the images of the Kaiser window function, dual- and tri-apodization, and those had 3.71, 3.64, 3.90 CNR value in order. This is summarized in Table 5.

**Table 5:** **CNR values of the singular window functions and dual-/tri-apodization**

	Rectangular window	Dolph-Chebyshev window	Kaiser window	Dual-apodization	Tri-apodization
CNR	2.58	4.26	3.71	3.64	3.90

## Discussion

In the point target simulation, the beam projection method capable of averaging the main-lobe width and the side-lobe level along the axial direction was employed to obtain more accurate results. The -6 dB main-lobe widths of dual- and tri-apodization were almost same as that of the rectangular window function. The -35 dB main-lobe widths of dual- and tri-apodization were 16.7 μm and 147.5 μm narrower than that of the rectangular window function at the focal point. In the case of point target simulation with a pulsed wave field, the boundary between main-lobe and side-lobe is not clear. Therefore, in order to compare the main-lobe widths of other windows or schemes, we defined -35 dB as a criterion considering -40 dB normal dynamic range in a B-mode image. In this level, some side-lobes of the rectangular window might be included as a main-lobe. Thus, -35 dB main-lobe width of rectangular window was broader than other schemes which have much smaller side-lobe level compared to the rectangular window. The side-lobe levels of dual-/tri-apodization were more suppressed by 9 ~ 10 dB. In the cyst target simulation, the Dolph-Chebyshev window had the highest CNR value and it was followed by tri-apodization, the Kaiser window, dual-apodization and the rectangular window in order. Because the side-lobe level of each window function was -13 dB, -50 dB, and -21 dB in order of the rectangular, Dolph-Chebyshev, and Kaiser window, the Dolph-Chebyshev window had the highest CNR value and the rectangular window had the lowest CNR value. In the case of dual-apodization, the first side-lobe level was similar with that of the rectangular window but the rest side-lobe level was about -50 dB. As a result, CNR value of dual-apodization existed between the rectangular window and Dolph-Chebyshev window. Additionally, since the highest side-lobe level of dual-apodization was higher than that of the Kaiser window, CNR value of the Kaiser window was followed by that of dual-apodization. In the case of tri-apodization, the highest side-lobe level was similar with that of the Kaiser window but the rest side-lobe level was lower than that of the Kaiser window until the Kaiser window has lower side-lobe level than that of the Dolph-Chebyshev window where the Kaiser window has -50 dB side-lobe level at the first time. Therefore, tri-apodization had higher CNR value than that of the Kaiser window. Consequently, CNR values of dual-/tri-apodization were 41% and 51% improved respectively compared with that of the rectangular window. Because dual- and tri-apodization are examples of the multi-apodization technique, the more window functions with lower side-lobe level used, the better CNR value can be obtained. However, this technique selects minimal value of images after applying different window functions, so the main-lobe pattern of added window function should cross the highest side-lobe resulting in suppression of the side-lobe. Thus, the control parameters determining the main-lobe width and the side-lobe level of each window function should be carefully chosen considering aforementioned issues. It has been well known that the first side-lobe level depends on the amplitude weighting function (apodization), and the high side-lobe called the grating lobe is affected by the element pitch size. Increasing the pitch size causes appearance of the grating lobe inside the view width. On the contrary, the lateral resolution affected by f-number (focal length/aperture size) is improved under the fixed number of elements in the subaperture. In this paper, we used the 128 element linear array transducer with 40 μm pitch. The pitch was slightly larger than one wave-length resulting in grating lobe appearance at 70 degree. However, the effects of the grating lobe can be negligible in this study due to the limited view width [18].

## Conclusions

In this study, the performance of the non-linear apodization was numerically investigated by applying to the high frequency ultrasound imaging. We demonstrated that dual- and tri-apodization can successfully suppress the side-lobe level without sacrificing lateral resolution resulting in increased CNR value. Therefore, dual- and tri-apodization can be one of the potential ways to effectively improve spatial and contrast resolution of a high frequency ultrasound B-mode image.
